# Treatment of disorders of gut–brain interaction with peppermint oil and caraway oil combination Menthacarin: A phase IV clinical trial

**DOI:** 10.1007/s10354-025-01119-2

**Published:** 2025-12-17

**Authors:** Ioannis Linas, Stephan R. Vavricka, Jost Langhorst, Daniel Pohl, Berenike Stracke, Andrea Zimmermann, Petra Funk, Ingolf Schiefke, Ahmed Madisch

**Affiliations:** 1 Gastroenterological Group Practice GGP, Bern, Switzerland; 2 Department of Gastroenterology and Hepatology, University Hospital Zürich, Zürich, Switzerland; 3 Sozialstiftung Bamberg, Klinikum am Bruderwald, Bamberg, Germany; 4 Global Medical Affairs, Dr. Schwabe Holding SE & Co. KG, Karlsruhe, Germany; 5 Research and Development, Dr. Willmar Schwabe GmbH & Co. KG, Karlsruhe, Germany; 6 Department of Gastroenterology, Hepatology, Diabetes Care, and Endocrinology, Klinikum St. Georg gGmbH, Leipzig, Germany; 7 Center of Gastroenterology Bethanien, Frankfurt, Germany; 8 Centre for Internal Medicine, DIAKOVERE Friederikenstift, Humboldtstr. 5, 30169 Hannover, Germany

**Keywords:** Abdominal symptoms, Stool consistency, Quality of life, Functional dyspepsia, Irritable bowel syndrome, Abdominelle Symptome, Stuhlkonsistenz, Lebensqualität, Funktionelle Dyspepsie, Reizdarmsyndrom

## Abstract

**Introduction:**

In disorders of gut–brain interaction including functional dyspepsia and irritable bowel syndrome, clinical focus has shifted from pathophysiological criteria to symptoms.

**Methods:**

A single-arm, low-intervention clinical trial into the effectiveness and tolerability of Menthacarin, a peppermint oil/caraway oil combination, was performed. A total of 126 patients with abdominal pain/cramps or a sensation of being bloated without organic cause received 1 capsule Menthacarin twice a day for 8 weeks. Assessments included abdominal symptoms, stool parameters, and quality of life.

**Results:**

During treatment, all assessed abdominal symptoms showed significant (*p* < 0.001), clinically meaningful improvements, with standardized effect sizes of 0.83–1.05 for change from baseline. The number of days/week with symptom impact or incomplete spontaneous bowel movements almost halved, while days/week with normal stool consistency increased (all *p* < 0.001). Health-related quality of life significantly improved (*p* < 0.001) and Menthacarin was well tolerated.

**Conclusion:**

The study demonstrates patient-relevant improvement in gastrointestinal symptoms during treatment with Menthacarin while underlining its favorable tolerability profile.

## Abstract (de)

**Hintergrund:**

Bei Darm-Hirn-Interaktionsstörungen, einschließlich funktioneller Dyspepsie und Reizdarmsyndrom, hat sich der klinische Schwerpunkt von pathophysiologischen Kriterien auf Symptome verlagert.

**Methoden:**

Es wurde eine einarmige, minimal-interventionelle klinische Prüfung zu Wirkungen und Verträglichkeit von Menthacarin, einer Pfefferminzöl-Kümmelöl-Kombination, durchgeführt. Dabei erhielten 126 Patienten mit abdominellen Schmerzen/Krämpfen oder Blähgefühl ohne organische Ursache 8 Wochen lang 2 × 1 Kapsel/Tag Menthacarin. Bewertet wurden abdominelle Symptome, Stuhlparameter und Lebensqualität.

**Ergebnisse:**

Während der Behandlung zeigten alle erhobenen abdominellen Symptome signifikante (*p* < 0,001), klinisch bedeutsame Verbesserungen, mit standardisierten Effektgrößen von 0,83–1,05 für Veränderungen gegenüber dem Ausgangswert. Die wöchentliche Anzahl Tage mit Symptomen oder unvollständigem Stuhlgang wurde nahezu halbiert (*p* < 0,001). Die wöchentliche Anzahl Tage mit normaler Stuhlkonsistenz nahm zu (*p* < 0,001). Signifikante Verbesserungen wurden auch bei der gesundheitsbezogenen Lebensqualität beobachtet (*p* < 0,001), und Menthacarin wurde gut vertragen.

**Schlussfolgerungen:**

Die Studie belegt eine patientenrelevante Verbesserung der gastrointestinalen Beschwerden unter Behandlung mit Menthacarin und unterstreicht dessen günstiges Verträglichkeitsprofil.

## Introduction

Disorders of gut–brain interaction (DGBI), formerly known as functional gastrointestinal (GI) disorders, are among the most frequent disorders seen in clinical practice. According to the Rome IV criteria published in 2016 [1], DGBI are characterized by GI symptoms related to any combination of motility disturbances, visceral hypersensitivity, altered mucosal and immune function, altered gut microbiota, or altered central nervous system (CNS) processing. The Rome IV definition represents a shift in clinical focus from pathophysiological criteria to symptoms. The focus on symptoms reflects the increasing recognition of the importance of the patients’ perspective within a patient-centered therapeutic model [2].

In a recent large-scale global study conducted by the Rome Foundation, the prevalence of functional dyspepsia (FD) and irritable bowel syndrome (IBS) was estimated to be 7.2% and 4.1%, respectively, based on the strict Rome IV criteria [3]. FD and IBS are assumed to share similar pathogenic mechanisms, including visceral hypersensitivity, impaired gastric accommodation, central hyperalgesia, impairment of gastrointestinal motility or epithelial barrier function, and/or alteration in the enteric nervous system and central processing [4, 5]. Evolving evidence suggests that not only gastric but also duodenal motility abnormalities and hypersensitivity are responsible for symptoms, mediated by complex mechanisms including low-grade inflammation, local immune processes, increased mucosal permeability, and/or altered duodenal content [6]. IBS is currently perceived to be related to disordered communication between the gut and the brain, with or without abnormal colonic motility or transit, abnormal intestinal or colorectal sensation, alterations in colonic bile acid concentration, the microbiome and discreet submucosal inflammatory processes, and abnormalities in serotonin metabolism [7, 8].

Studies investigating the overlap between IBS and FD as diagnosed according to the Rome IV criteria found comorbidity rates exceeding 50% [9, 10]. Patients with overlapping FD and IBS were found to have more severe symptoms and to be more difficult to treat compared to those with one of the conditions alone [9, 11]. FD and IBS, like most other DGBI, are associated with poor quality of life, as well as more frequent and more intensive use of healthcare resources, also resulting in a considerable economic burden for both patients and society [3, 12, 13].

One of the objectives of the Rome IV classification was to categorize patients into symptom-based subclasses, enabling targeted treatment based on the assumption of similar underlying pathophysiology. However, the overlap of symptoms between different DGBI—such as FD and IBS—may adversely affect treatment outcomes due to a lack of specific guidance on managing overlapping DGBI [14]. Recently, a guideline on the management of FD focusing on overlapping GI symptoms was published by a working group of the Asian Pacific Association of Gastroenterology [14]. The authors proposed four overlapping symptom clusters. One of these clusters is postprandial distress syndrome with IBS, characterized by fullness, bloating, belching, and flatulence. Treatment options recommended for this symptom cluster include herbal medicines, which are considered attractive by the authors due to their favorable tolerability profile and their prospect to target multiple pathophysiological mechanisms simultaneously [15]. A specific recommendation is given for a combination of peppermint oil and caraway oil [14]. This recommendation is in line with the recently published German S1 guidelines on functional dyspepsia, which summarize the current state of knowledge and consider peppermint oil in combination with caraway oil to be a first-line therapy that alleviates the symptoms of FD and can be considered for FD patients [16]. Moreover, the German Consensus Guidelines on Definition, Pathophysiology and Management of IBS recommend peppermint oil in various forms of preparation for the treatment of the IBS-associated symptoms pain and flatulence [17].

Menthacarin^1^ is a proprietary combination of peppermint oil (90 mg WS^®^ 1340) and caraway oil (50 mg WS^®^ 1520) with specified quality, which is marketed as a medicinal product in several countries. Peppermint oil has an analgesic and spasmolytic effect by blocking pain-mediated signals via activation of cold receptors (TRPM8) and relaxation of smooth muscle cells through inhibition of voltage-dependent Ca^2+^ channels [18–20]. Caraway oil, on the other hand, has an antimeteoric and foam-reducing effect mediated by a reduction in surface tension and a decrease in gas formation that also has a beneficial impact on the gut microbiome [5, 21]. For the combination of the two essential oils, a reduction of visceral hypersensitivity has been shown [5, 22]. Specifically, in a rat model, Menthacarin was found to reduce the net excitatory response of anterior cingulate cortex neurons to colorectal distension and to counteract the development of visceral hyperalgesia and hypersensitivity [23]. These effects were observed during chronic administration of the product but not for acute administration. Three recent meta- and subgroup analyses based on evidence from randomized, controlled clinical trials have demonstrated that Menthacarin is efficacious in both FD and IBS as well as in patients with an overlap of both conditions [5, 24, 25].

We report on a phase IV clinical trial performed to systematically assess changes in DGBI-associated symptoms during treatment with Menthacarin, under conditions resembling those in real-world clinical practice. The study also explored the effects of the herbal combination on patient-relevant outcomes such as quality-of-life and treatment satisfaction.

## Methods

### Overview and ethical conduct

This multicenter, uncontrolled, phase IV clinical trial was a low-intervention trial, as defined by the European Union Clinical trials Regulation (EU 536/2024). Patients received 1 capsule Menthacarin twice a day for 8 weeks, preceded by a 7-day, medication-free screening period. Visits were scheduled at start of screening, at end of screening (baseline), as well as after 28 and 56 days of treatment.

The protocol was reviewed and approved by the competent authorities and the competent independent Ethics Committees. The principles of Good Clinical Practice and the Declaration of Helsinki, as amended in October 2013, were adhered to. The study was registered in the European Union Clinical Trials Register, EU CT no. 2022-501800-92-00, and in the Registry of all Projects in Switzerland (RAPS), BASEC-ID 2023-00383. All participants provided written informed consent.

### Participants

The trial was performed in private practices and medical institutions in Germany and Switzerland between June 2023 and January 2024. Investigators had to be general practitioners, specialists in internal medicine, or gastroenterologists experienced in the treatment of DGBI.

The symptom picture of the complaints defined by the inclusion and exclusion criteria ensured that the use of the investigational medicinal product followed the terms of the authorization: patients had to be suffering from abdominal pain/cramps and/or a sensation of being bloated on at least 1 day per week for at least 3 months. Moreover, at least 1 of the following 3 functional symptoms had to be present on at least 1 day during the last week before the first screening visit (retrospective assessment), with an intensity of ≥ 4 points (numerical rating scale ranging from 0–10): abdominal pain/cramps, sensation of being bloated, or postprandial fullness. Subsequently, at least 1 of the functional abdominal symptoms for which an intensity of ≥ 4 points had been observed prior to the start of screening had to be present again with an intensity of ≥ 4 points on at least 1 day during the last week prior to the end-of-screening visit (prospective assessment by daily diary) to confirm eligibility.

Specific exclusion criteria were abdominal symptoms attributable to an organic cause, constipation, structural gastrointestinal (GI) tract abnormalities or diseases affecting bowel transit, bloody diarrhea, achlorhydria, and diseases of the biliary system or liver. Patients with known hypersensitivity to peppermint, menthol, caraway, or other ingredients of the investigational product were also excluded. Concomitant use of any treatments for DGBI other than the investigational product, including herbal preparations, was not allowed.

### Intervention

Menthacarin was provided in gastro-resistant soft capsules for oral intake. One gastro-resistant soft capsule contained 90 mg Mentha × piperita L., aetheroleum (peppermint oil) and 50 mg Carum carvi L., aetheroleum (caraway oil). One capsule twice a day was to be taken unchewed 30 min before breakfast and lunch, respectively, with an interval of at least 1 h to the intake of other medicinal products administered to decrease stomach acid, such as antacids, proton pump inhibitors, or histamine‑2 blockers. The first capsule was to be taken in the morning on the day following the baseline visit.

### Assessments

Between screening and end of treatment, patients had to record the maximum intensity of the following symptoms experienced during the last 24 h in a diary on a daily basis, using numerical rating scales ranging from 0 (absent) to 10 (maximum intensity): “abdominal pain/cramps”, “sensation of being bloated (i.e., feeling pressure or fullness)”, “bloated appearance (i.e., belly swollen)”, “flatulence (i.e., passing gas)”, “abdominal sounds (i.e., gurgling or rumbling)”, and “postprandial fullness”. A total score was calculated by summing up the individual symptom scores during statistical analysis. Moreover, participants had to identify which of the three given abdominal symptoms (abdominal pain/cramps, sensation of being bloated, or postprandial fullness) was the most bothersome. The diary also included daily assessments of the total number of spontaneous bowel movements (SBM) as well as the number of incomplete SBM, of stool consistency (using the Bristol Stool Form Scale [26]), and a global question about the impact of the symptoms (“Do you feel affected by your abdominal symptoms?” [yes/no]). Furthermore, the dates of the first and last intake of the investigational product were recorded.

Patients had to identify the most bothersome abdominal symptom at each site visit. During post-baseline visits, patients were asked about their satisfaction with changes in bowel habits. They were also asked to provide separate, global assessments of the effectiveness and tolerability of treatment, and to complete the Integrative Medicine Patient Satisfaction Scale (IMPSS) [27]. Health-related quality of life (QoL) was assessed at both baseline and the end of treatment using the Short Form 12 Health Survey (SF-12 version 2.0; German version) [28, 29]. SF-12 is a self-reported survey of patient health and well-being, which consists of 12 questions divided in eight sections (vitality, physical functioning, bodily pain, general health perceptions, physical role functioning, emotional role functioning, social role functioning, and mental health). The results are represented by two scores: one for the physical component and one for the psychological component, each ranging between 0 and 100 points. The SF-12 uses a norm-based rating system, where, for each component, the mean ± standard deviation for the general population is 50 ± 10 points, indicating average QoL [29]. Scores higher than this indicate a better QoL, while lower scores are indicative of a poorer QoL. Global assessments of effectiveness and tolerability were also obtained from the investigators at both post-baseline visits. Moreover, the investigators had to question the patients about any adverse events.

### Statistics

In accordance with the single-arm design of the study, a descriptive statistical concept was applied. Outcomes rated daily in the patient diary were analyzed based on weekly averages, which were also used to compute the change from baseline. In this context, the baseline values were determined as the mean values of the daily ratings from the week before the start of treatment, and the values for week 8 were computed as the averages of the ratings obtained during the week before the scheduled treatment end. Analyses of change between baseline and week 8 (scheduled end of treatment) were based on 95% confidence intervals (CI) and t‑test statistics, including descriptive *p*-values for the null hypothesis predicting no change from baseline. Standardized effect size estimates were computed by dividing the means for intraindividual change between baseline and week 8 by the corresponding standard deviations. Additionally, responder analyses were performed, considering a symptom decrease of at least 30% of the baseline value as a clinically meaningful change, as per applicable regulatory guidance [30, 31]. All data were analyzed descriptively.

Patients who terminated their participation in the study before week 8 and patients without valid diary data for week 8 were excluded from the analysis of change between baseline and week 8. Thus, sensitivity analyses were performed for the same endpoints, based on change between baseline and the last individual post-baseline visit.

All patients who had received at least one dose of the investigational product were assessed for safety. All patients from the safety set who provided baseline data and any post-baseline data for at least one outcome measure were analyzed for effectiveness (full analysis set). Analyses were based on observed data, i.e., no missing data imputation was performed.

The sample size estimation was based on an average 1‑point difference between baseline and end of treatment, with a standard deviation of 3.3 points (i.e., a standardized effect size of 0.3) on the 11-point numerical rating scales. Assuming a correlation of 0.4 between the values at baseline and end of treatment, a sample size of at least 107 patients was expected to be required to ensure at least 80% power.

## Results

### Participant accountability and characteristics

**Fig. 1:**
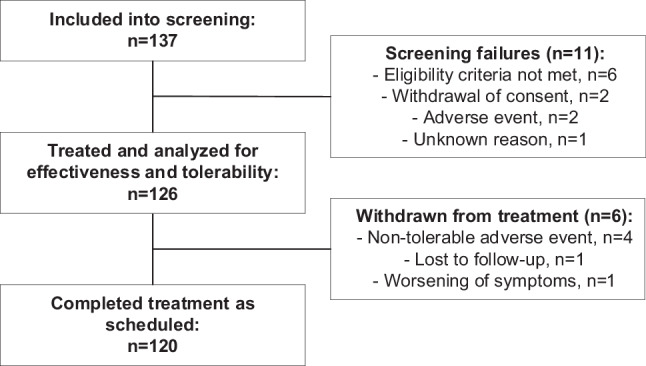
Disposition of patients

A total of 137 patients were included in medication-free screening in 13 sites in Germany and Switzerland, and 126 were treated with Menthacarin and analyzed for effectiveness and safety. Of these, 120 eligible participants (95.2%) completed treatment as scheduled after 8 weeks (Fig. 1).

The study participants were between 18 and 81 years of age; more than 3/4 were female (Table 1). All participants were Caucasians, even though patients of any ethnic origin had been eligible for inclusion.

**Table 1:** Baseline characteristics (*n* = 126; means ± standard deviations or absolute numbers and proportions of patients)

Age (years)		46.1 ± 17.0
Sex	Female	99 (78.6%)
	Male	27 (21.4%)
Height (cm)		170.2 ± 8.6
Weight (kg)		74.8 ± 16.4
Current gastrointestinal disorder (other than the study indication)		21 (16.7%)
Leading symptoms at baseline	Abdominal pain/cramps	122 (96.8%)
	Sensation of being bloated	124 (98.4%)
Most bothersome abdominal symptom	Abdominal pain/cramps	46 (36.5%)
	Sensation of being bloated	69 (54.8%)
	Postprandial fullness	11 (8.7%)

At baseline, the most bothersome abdominal symptom was a sensation of being bloated, which was named by more than half of the study participants, followed by abdominal pain/cramps and postprandial fullness.

At study inclusion, 83 patients (65.9%) suffered from concurrent disorders among which hypertension (*n* = 30, 23.8%), asthma (*n* = 14, 11.1%), hypothyroidism (*n* = 14, 11.1%), and hypercholesterolemia (*n* = 11, 8.7%) were the most frequent. Among the concurrent GI disorders, gastroesophageal reflux disease (*n* = 9, 7.1%), intestinal diverticulum (*n* = 6, 4.8%), and hemorrhoids (*n* = 4, 3.2%) were reported most frequently. Ninety patients (71.4%) received any concomitant medication, the most frequent of which were antihypertensives.

The amount of investigational product taken, as calculated based on the patient diary entries, corresponded to an average of 98.1% ± 9.9% (mean ± standard deviation) of the prescribed amount.

### Abdominal symptoms

**Fig. 2:**
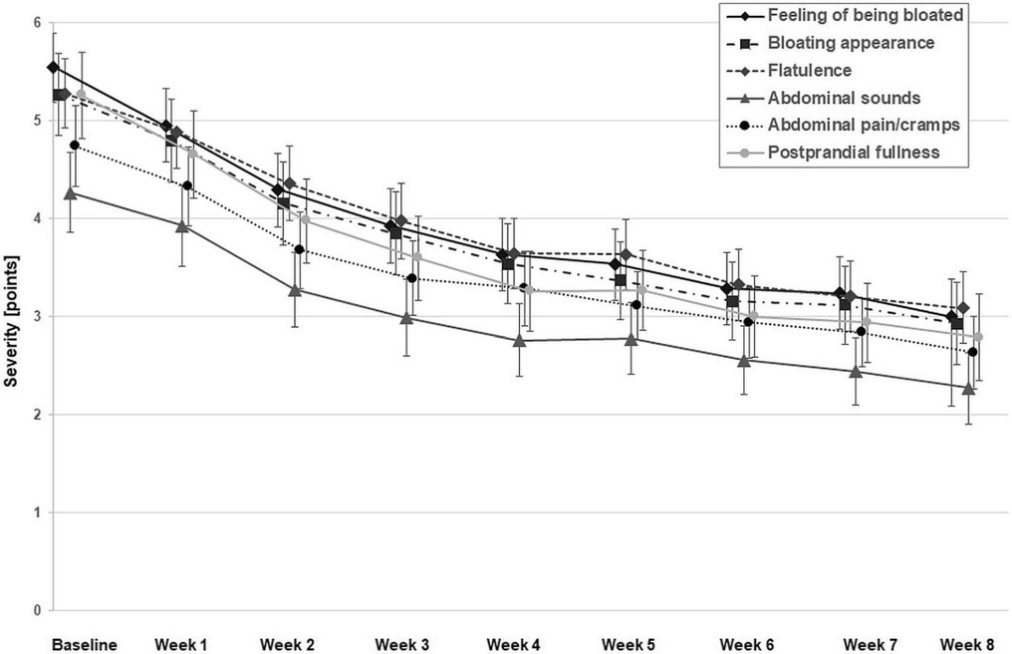
Course of severity of abdominal symptoms during treatment with Menthacarin (weekly means and 95% confidence intervals; baseline, *n* = 126; Week 1, *n* = 125; Weeks 2 to 4, *n* = 124; Week 5, *n* = 122; Week 6, *n* = 120; Week 7, *n* = 121; Week 8, *n* = 116)

**Fig. 3:**
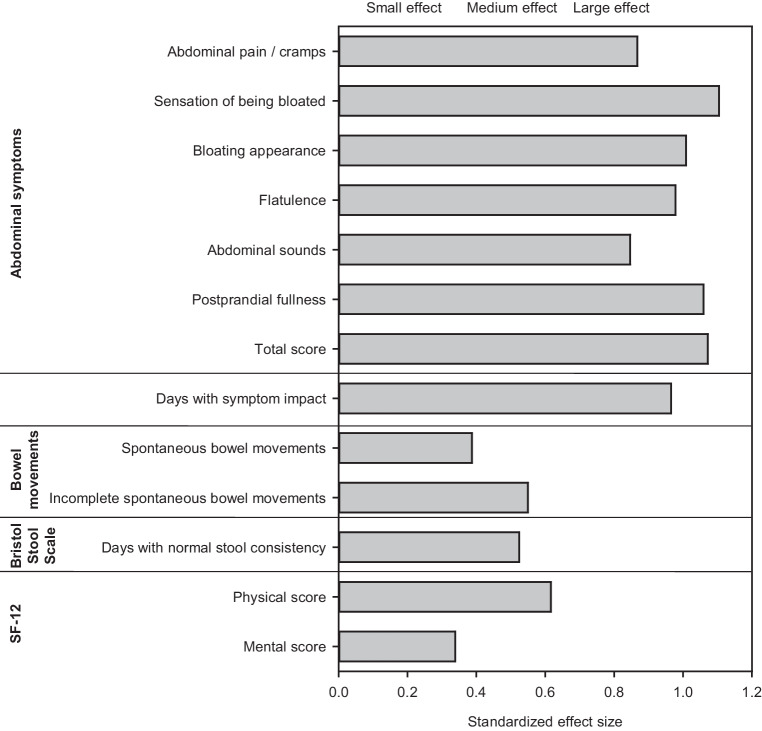
Effectiveness outcomes—standardized effect size for change between baseline and week 8

Based on the self-ratings in the patient diary, the most severe abdominal symptom at baseline was sensation of being bloated, followed by bloating appearance, flatulence, postprandial fullness, abdominal pain/cramps, and abdominal sounds (i.e., borborygmi). During treatment with Menthacarin, all symptoms improved steadily until the end of the observation period (Fig. 2). Mean intraindividual differences ranged between 2.0 points for abdominal sounds and 2.5 points for sensation of being bloated and postprandial fullness (Table 2), while standardized effect size estimates ranged between 0.85 for abdominal sounds and 1.10 for sensation of being bloated (Fig. 3). According to the conventional interpretation of standardized effect sizes [32], the changes observed for all abdominal symptoms and for the total score (d = 1.07) corresponded to a large effect.

**Table 2:** Effectiveness outcomes (weekly averages)—baseline value (mean ± standard deviation), change between baseline and week 8 (mean, 95% confidence interval, t‑test *p*-value), as well as absolute numbers and proportions of patients with a ≥ 30% improvement between baseline and the last individual assessment

Symptom/Parameter	Baseline value (*n* = 126)	Change from baseline to week 8 (*n* = 116* unless otherwise indicated)	*p*	Patients improved by ≥ 30% (*n* = 126)
Abdominal symptoms**				
Abdominal pain/cramps	4.7 ± 2.4	−2.1 (−2.5, −1.7)	< 0.001	78 (61.9%)
Sensation of being bloated	5.6 ± 2.0	−2.5 (−3.0, −2.1)	< 0.001	81 (64.3%)
Bloating appearance	5.3 ± 2.4	−2.3 (−2.8, −1.9)	< 0.001	73 (57.9%)
Flatulence	5.3 ± 2.0	−2.2 (−2.7, −1.8)	< 0.001	72 (57.1%)
Abdominal sounds	4.3 ± 2.3	−2.0 (−2.4, −1.6)	< 0.001	80 (63.5%)
Postprandial fullness	5.2 ± 2.5	−2.5 (−2.9, −2.1)	< 0.001	81 (64.3%)
Total score	30.4 ± 11.7	−13.7 (−16.0, −11.4)	< 0.001	78 (61.9%)
Number of days per week with symptom impact**	6.1 ± 1.6	−2.8 (−3.4, −2.3)	< 0.001	–
Bowel movements per day**				
Spontaneous bowel movements	2.2 ± 1.5	−0.4 (−0.5, −0.2)	< 0.001	–
Incomplete spontaneous bowel movements	1.0 ± 1.1	−0.5 (−0.7, −0.3) (*n* = 115)	< 0.001	–
*Bristol Stool Form Scale*				
Stool consistency***	4.5 ± 1.3 (*n* = 123)	−0.4 (−0.6, −0.1) (*n* = 108)	0.008	–
Number of days per week with normal stool consistency****	3.3 ± 2.2	+1.5 (+1.0, +2.0)	< 0.001	–
SF-12****				
Physical score	58.1 ± 17.2	+10.7 (+7.5, +14.0)	< 0.001	–
Mental score	58.2 ± 17.0	+5.5 (+2.5, +8.5)	< 0.001	–

* For most of the diary-based effectiveness outcomes presented, the number of evaluable participants is 116 because 4 of the patients who completed the study as scheduled failed to maintain their diary until week 8

** negative changes are indicative of an improvement

*** range: 1 (hard stool) – 7 (watery diarrhea)

**** positive changes are indicative of an improvement

Between baseline and individual treatment end, a clinically meaningful treatment effect (defined by a ≥ 30% symptom score decrease compared to baseline) was observed in between 57.1% (flatulence) and 64.3% (sensation of being bloated, postprandial fullness) of the patients (Table 2).

The average number of days per week on which patients felt affected by abdominal symptoms decreased from 6.1 ± 2.8 days at baseline to 3.3 ± 3.0 days at week 8, corresponding to a difference of −2.8 ± 2.9 days (*p* < 0.001) and a standardized effect size of 0.97. All of the assessed abdominal symptoms improved (*p* < 0.001). The largest improvements and effect sizes, as well as the greatest proportion of patients improving by ≥ 30% during treatment with Menthacarin were observed for sensation of being bloated and postprandial fullness (Table 2; Fig. 3).

### Spontaneous bowel movements and bowel habits

During treatment with Menthacarin, the average number of SBMs per day decreased from 2.2 ± 1.5 (baseline week) to 1.9 ± 1.2 (Week 8) for all SBMs and from 1.0 ± 1.1 (baseline week) to 0.5 ± 0.8 (Week 8) for incomplete SBMs, i.e., the number of incomplete SBMs was reduced by approximately 50% (*p* < 0.001 each), with standardized effect sizes of 0.39 (all SBMs) and 0.55 (incomplete SBMs), respectively (Table 2; Fig. 3).

The number of patients expressing that their bowel habits had changed to their satisfaction increased from 56 (44.4%; 95% confidence interval [CI] 35.8–53.1%) after 4 weeks of treatment to 73 (57.9%; 95% CI 49.3–66.6%) at the end of week 8.

### Stool consistency

Stool consistency was assessed based on the Bristol Stool Form Scale. During the observation period, a slight shift in the mean towards a more solid stool consistency was observed, coinciding with an increase in the number of days per week with normal stool consistency (*p* < 0.01; effect size: 0.52; Table 2; Fig. 3).

### Health-related quality of life

The responses in the SF-12 health survey showed significant improvements (*p* < 0.001) in the patients’ health-related QoL during treatment with Menthacarin (Table 2; Fig. 3). Improvements were more pronounced in the physical health domain (effect size: 0.62) than in the mental health domain (effect size: 0.34).

### Global effectiveness assessments

**Fig. 4:**
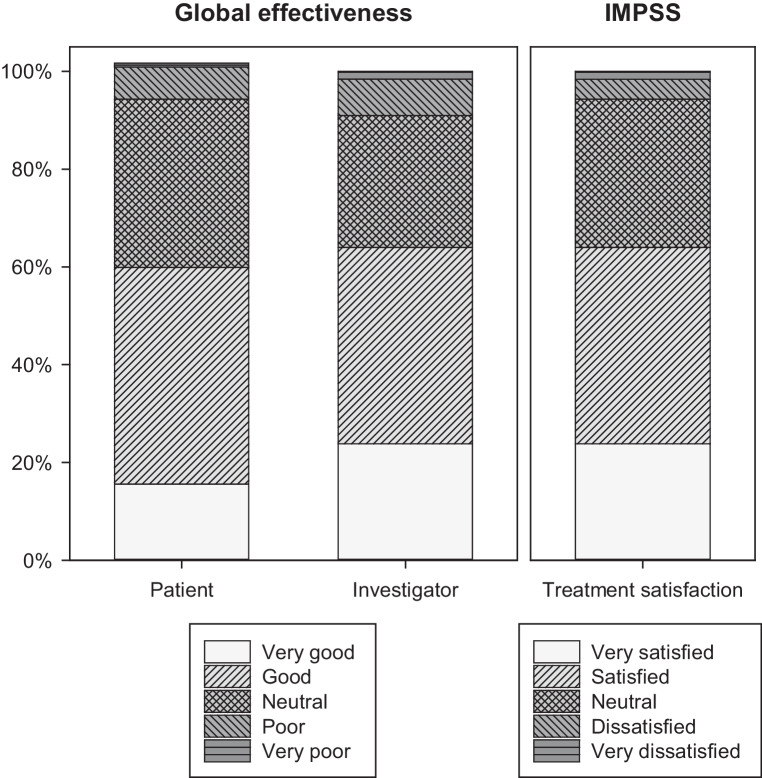
Global assessment of treatment effectiveness and Integrative Medicine Patient Satisfaction Scale (IMPSS)—ratings at week 8 (proportion of patients based on *n* = 122 patients with valid data)

According to the IMPSS assessment at the end of week 8, 49 patients (40.2%) stated that they were satisfied with the investigational treatment and 29 (23.8%) stated that they were very satisfied (Fig. 4; basis: 122 patients with valid data at week 8). Accordingly, global assessment of treatment effectiveness was rated as ‘good’ by 54 (44.3%) and as ‘very good’ by 19 (15.6%) patients. Similar global assessments were provided by the investigators who rated the effectiveness of the treatment as ‘good’ in 49 patients (40.2%) and as ‘very good’ in 29 patients (23.8%). Patient satisfaction and global ratings concerning treatment effectiveness at week 8 were slightly more favorable than those obtained at week 4 (data not shown). In all, 88 patients (72.1% of 122 patients with valid data) affirmed their willingness to recommend Menthacarin.

### Sensitivity analyses

Sensitivity analyses performed for the endpoints based on change between baseline and the last individual post-baseline visit showed that differences to the primary analysis were marginal and did not lead to other conclusions (data not shown).

### Tolerability

During the 8‑week treatment with Menthacarin, 21 patients (16.7%) reported any adverse events. The most frequent events were infections (11 patients, 8.7%), followed by gastrointestinal disorders (7 patients, 5.6%). The only events that occurred in more than one patient were COVID-19 infection (*n* = 3) and unspecified viral infection (*n* = 3), as well as abdominal pain (*n* = 2), gastrointestinal reflux disease (*n* = 2), and headache (*n* = 2). Six patients (4.8%) had seven adverse events the causal relationship of which with the investigational treatment was assessed as unlikely (reflux, *n* = 2; worsening of abdominal pain, *n* = 1; headache, *n* = 2; diverticulitis, *n* = 1; and worsening of hypertension, *n* = 1). All other events were assessed to be unrelated. One serious adverse event (i.e., an infection of a knee endoprosthesis that necessitated hospitalization and surgery) was considered evidently related to a known cause other than Menthacarin. In four cases, the adverse event led to the patient’s withdrawal from treatment (diverticulitis, *n* = 1; knee endoprosthesis, *n* = 1; worsening of abdominal pain, *n* = 1; reflux, *n* = 1).

Global assessments of tolerability (week 8) were available for 122 patients. Of these patients, 69 (56.6%) rated the tolerability of Menthacarin as ‘very good’ and 43 (35.2%) as ‘good’, while 1 patient (0.8%) rated it as ‘very poor’. According to the investigators’ ratings, 119 patients (97.5%) tolerated Menthacarin well or very well, while no negative tolerability ratings were obtained.

## Discussion

With the introduction of the Rome IV criteria, the focus in DGBI shifted from indications to clusters of symptoms [2]. Moreover, the focus on symptoms reflects the increasing recognition of the importance of the patients’ perspective within a patient-centered therapeutic model [33–35].

This low-intervention clinical trial was performed in patients presenting with the DGBI-related symptoms abdominal pain/cramps, sensation of being bloated, or postprandial fullness without organic cause. There was a clear focus on symptoms and other patient-reported outcomes. The symptom picture of the complaints, as defined by the inclusion and exclusion criteria, ensured that the use of the investigational medicinal product was in accordance with the terms of the authorization. The results demonstrate consistent and clinically substantial improvements in the investigated abdominal symptoms (abdominal pain/cramps, sensation of being bloated, bloating appearance, flatulence, abdominal sounds, postprandial fullness) during the 8‑week treatment with Menthacarin. This was especially true in those symptoms perceived as most bothersome by the majority of patients. Symptom alleviation was observed for both gastric and intestinal complaints, including a substantial increase in the number of days per week with normal stool consistency. Moreover, the reduction in complete spontaneous bowel movements and the halving of incomplete ones may provide particular relief for patients. This may be an important contribution to reducing disease-associated distress in everyday situations.

This interpretation is supported by the results of the SF-12 QoL assessment. Although baseline mean values of the mental and physical scores were already somewhat better than the mean values seen in the general population [29], the scores improved further during treatment. This shows that the relief of GI symptoms led to noticeable improvements in the patients’ everyday lives. The fact that the number of days per week on which the patients felt impaired by their GI symptoms was almost halved under treatment with Menthacarin may also have contributed to this. The results are also consistent with the global assessments of treatment satisfaction and effectiveness, which show that more than 60% of the study participants were satisfied or very much satisfied with the treatment, combined with a high degree of willingness to recommend Menthacarin to other patients with similar complaints.

Looking at the results of previous studies that can be compared based on the use of similar outcomes, the following can be stated: For symptoms of pain, pressure, heaviness, and fullness, the changes between baseline and the end of week 4 observed in this study (Fig. 2) were in the range of the results for Menthacarin in several randomized, placebo- or reference-controlled 4‑week trials in patients with FD and/or IBS [36–40]. The present study also shows that the alleviations of DGBI symptoms observed during the initial 4 weeks of treatment with Menthacarin were not only sustained, but most patients continued to improve until the end of the 8‑week observation period. Similar results were observed in the 11-month follow-up study reported by Storr and Stracke [41], following a placebo-controlled 4‑week trial reported by May et al. [38]: Improvement of symptoms achieved in patients treated with Menthacarin was sustained or even intensified over the full study period. During follow-up, patients who had initially been randomized to placebo were also switched to Menthacarin and, after about 6 months, achieved a comparable improvement to those who had initially been assigned to the herbal product. Another placebo-controlled 4‑week trial [40] investigated the efficacy of Menthacarin in patients with FD, using the Nepean Dyspepsia Index (NDI [42]) as the primary outcome for efficacy. During the double-blind 4‑week follow-up period (total treatment duration: 8 weeks), the Menthacarin group showed further improvement in the NDI total score, which was significantly superior to the placebo group [7]. Thus, together with the two follow-up studies described above, this study supports the effectiveness of Menthacarin in DGBI beyond the 4‑week period investigated in randomized, controlled trials.

The very favorable tolerability of Menthacarin is underlined by the fact that only 6 patients out of the 126 treated reported adverse events in which a causal relationship with Menthacarin could not be entirely ruled out. This is consistent with both the Summary of Product Characteristics (SmPC) and observations from other clinical trials in which the rate of adverse events observed under Menthacarin was consistently on a similar level to placebo treatment [5, 24, 25]. The favorable tolerability assessment is also supported by the high adherence rate observed in this study.

Strengths of the study include a clear focus on symptoms and other patient-relevant outcomes, including a detailed assessment of the important symptom complex of bloating (sensation of being bloated, bloating appearance, flatulence, borborygmi [‘abdominal sounds’]), for which a significant alleviation of the disease burden was achieved, a broad study population of patients affected by functional abdominal complaints, and a treatment situation close to ‘real-world’ setting. A limitation was the uncontrolled study design. This was chosen to ensure proximity to routine treatment outside of clinical trials, but it does not, in principle, allow causal inferences to be drawn about the magnitude of treatment effects. The observed effects could be influenced by confounding factors, e.g., placebo effects or regression to the mean, which make it difficult to isolate the true effect of the treatment. On the other hand, the improvements in symptoms observed during the course of the investigational treatment can hardly be plausibly explained by the natural course of the disease or as a consequence of a reactive study design in this low-intervention trial. Moreover, the observed effects were consistent with those in former placebo- and reference-controlled clinical trials with Menthacarin in FD and IBS [5, 25], as well as with follow-up studies investigating the long-term effects of the herbal product [7, 41]. Taken together, the chosen low-interventional study design proved suitable for investigating treatment with Menthacarin in a real-world setting without compromising on the important outcomes or clinical research quality standards.

In conclusion, the majority of participants in this low-intervention trial experienced clinically relevant improvements in DGBI-associated complaints while undergoing Menthacarin treatment. These improvements were highly consistent across abdominal symptoms, stool parameters, and other effectiveness outcomes. Moreover, considerable improvements in health-associated quality of life were observed, underlining the patient-relevance of the results. The study also supports the favorable tolerability profile of Menthacarin, which may thus be a valuable option in the symptomatic treatment of DGBI, contributing to the reduction of disease-associated distress in everyday situations of patients.

## Footnotes

1 Menthacarin® is the active substance of Carmenthin® and Gaspan®, manufactured by Dr. Willmar Schwabe Pharmaceuticals, Karlsruhe, Germany.
